# The Northern Root-Knot Nematode *Meloidogyne hapla*: New Host Records in Portugal

**DOI:** 10.3390/biology11111567

**Published:** 2022-10-26

**Authors:** Leidy Rusinque, Filomena Nóbrega, Clara Serra, Maria L. Inácio

**Affiliations:** 1 Instituto Nacional de Investigação Agrária e Veterinária (INIAV, I.P.), 2780-159 Oeiras, Portugal; 2 Centre for Functional Ecology-Science for People & the Planet, Department of Life Sciences, University of Coimbra, Calçada Martim de Freitas, 3000-456 Coimbra, Portugal; 3 Direção-Geral de Alimentação e Veterinária, DGAV, 1349-017 Lisboa, Portugal; 4 GREEN-IT Bioresources for Sustainability, ITQB NOVA, Av. da República, 2780-157 Oeiras, Portugal

**Keywords:** identification, SCAR markers, sequencing, phylogenetic

## Abstract

**Simple Summary:**

Damage caused by plant-parasitic nematodes (PPNs) is usually underestimated as many farmers are oblivious of their presence. Root-knot nematodes (RKNs) are an economically important group of PPNs and one of the most devastating. The species *Meloidogyne arenaria*, *M. hapla*, *M. incognita* and *M. javanica* are considered the most important, due to their worldwide distribution and polyphagia, being the northern RKN, *Meloidogyne hapla* the most important species occurring in cold regions. During 2019–2022, surveys were carried out in different districts of Portugal in horticultural and ornamental crops. From the samples collected, nine were identified morphologically and molecularly as *M. hapla*, indicating that its prevalence is increasing, and demonstrating its importance and impact in agricultural systems.

**Abstract:**

Root-knot nematodes (RKNs), *Meloidogyne* spp., are a group of plant-parasitic nematodes (PPNs) of great economic significance worldwide. The northern root-knot nematode, *Meloidogyne hapla*, is one of the most important species of RKNs occurring in cold regions. In Portugal so far, *M. hapla* has been found parasitizing potato and fig trees. During surveys carried out in 2019–2022 in fields for horticultural and ornamental production, soil and root samples were collected. Roots were observed under a stereomicroscope to determine the presence of galls. Nematodes were extracted from the soil. Morphological features showed a high similarity and consistency with previous descriptions of the genus. For molecular analysis, total genomic DNA was isolated from single nematodes and used to amplify in multiplex reaction using the species-specific primers JMV1, JMV2 and JMVhapla, and for sequencing of the ITS region with the primers TW81/AB28. Multiplex PCR amplification produced a specific fragment of 440 bp and PCR amplification of the ITS region yielded a single fragment of 550 bp, as expected. The obtained sequences showed a similarity ranging from 99.8% to 100% with the sequences of *M. hapla* available in the database. The phylogenetic tree revealed that the isolates grouped with *M. hapla* isolates. From the 690 samples collected, *M. hapla* was detected in three different hosts (grapevine, eucalyptus and potato) in four districts of mainland Portugal and on Madeira Island. To our knowledge, this is the first report of *M. hapla* infecting the grapevine and eucalyptus in Portugal.

## 1. Introduction

Horticulture crops represent about 50% of the Portuguese agricultural added value. The most important crops are grapevines, oranges, apples, pears, cole crops, peaches, processing tomatoes and potatoes. Ornamentals (cut flowers and potted plant production) in increasing areas represent about 600 ha [1].

Plant-parasitic nematodes (PPNs) are an important constraint to agricultural production as the losses they cause have been estimated from USD 175 billion per year [2,3]. However, the full extent of nematode damage is likely to be underestimated as many growers are unaware of their presence [4]. 

Root-knot nematodes (RKNs) are one of the oldest known parasitic nematodes of plants and considered serious pests of economically important crops [5]. The genus comprises more than 100 species, with many of them reported in Portugal: *Meloidogyne arenaria* (Neal, 1889) Chitwood, 1949; *Meloidogyne chitwoodi* Golden et al., 1980; *Meloidogyne enterolobii* Yang and Eisenback, 1983; *Meloidogyne hapla* Chitwood, 1949; *Meloidogyne hispanica* Hirschmann, 1986; *Meloidogyne incognita* (Kofoid and White, 1919) Chitwood, 1949; *Meloidogyne javanica* (Trub, 1885) Chitwood, 1949; *Meloidogyne luci* Carneiro et al., 2014; *Meloidogyne lusitanica* Abrantes and Santos, 1991; and *Meloidogyne naasi* Franklin, 1965 [6,7,8,9,10,11,12]. The species, *M. arenaria*, *M. hapla*, *M. incognita* and *M. javanica*, are regarded as the most important, due to their worldwide distribution and polyphagia [2,4]. Typical symptoms of RKN attack include galling of the root system, stunting and yellowing, resulting in impaired root function which leads to a reduction in yield [13,14].

The northern RKN, *Meloidogyne hapla,* was first described from a potato field in Long Island, New York (Chitwood 1949) and is one of the most important species of RKNs occurring in temperate regions, as it can withstand the cold. Eggs and juveniles can survive field temperatures below 0 °C, with some studies recording survival down to −15 °C in soil for a prolonged period of time. The optimum temperature for penetration and development of *M. hapla* is in the range of 20–25 °C and a mean temperature of 28 °C may prevent its development [15,16,17]. *Meloidogyne hapla* is a sedentary, biotrophic parasite of plants that overwinters in soil or diseased roots, and multiplies by both sexual (amphimixis) and asexual (parthenogenesis) reproduction, in contrast to the most widespread, three other RKNs [18]. Unlike many other RKN species, *M. hapla* eggs and juveniles can survive field temperatures below 0 °C. However, there is no evidence of its inability to survive in hot temperatures [17]. It parasitizes nearly all temperate vegetables of economic importance, reducing considerably yield and even causing total crop losses [14,19]. Surveys of vineyards and potatoes conducted in the United States found *M. hapla* to be the most abundant RKN present in the fields [20]. In Portugal, *M. hapla* was first detected in 2008 in fig trees and a year later found parasitizing the potato; since then, no other detections have been reported [7,8].

Therefore, the aim of the present study was to morphologically and molecularly identify the isolates of the RKN found in the survey of different counties and crops across mainland Portugal and Madeira Island, namely in grapevines, potato and eucalyptus.

## 2. Materials and Methods

### 2.1. Sampling and Nematode Isolates

During 2019–2022, surveys of horticultural and ornamental fields were carried out, resulting in a total of 690 soil and root samples collected from various districts in Portugal. Soil sampling was carried out from the rhizosphere at about 15 cm in depth for horticultural crops and 70 cm for grapevines and trees. Each sample consisted of 5 to 8 cores, sampled in the vicinity of plants that presented symptoms, and in zigzag at roughly equal intervals in asymptomatic fields. Each composite soil sample was placed in a polyethylene bag and brought for analysis. A 400-mL subsample was taken from each composite sample and the nematodes were extracted according to the protocol PM 7/119 (1) [21]. The suspension was observed under a stereomicroscope (Nikon SMZ1500, Tokyo, Japan) and suspected specimens of *Meloidogyne* observed using a bright-field light microscope (Olympus BX-51, Hamburg, Germany) for confirmation. When second-stage juveniles (J2) of *Meloidogyne* were detected in the soil suspension, bioassays were carried out by planting tomato plants cv. Oxheart in the remaining soil from the sample and maintained in a quarantine greenhouse for two months to obtain different developmental stages for further studies. Roots (from sampling and bioassays) were observed under stereomicroscope. Egg masses were handpicked from infected roots and used to establish cultures of each isolate.

### 2.2. Morphological Characterization

Ten second-stage juveniles (J2) from each positive sample were placed in a drop of water on a glass slide, gently heat killed for morphological characterization using a bright-field light microscope (Olympus BX-51, Hamburg, Germany) and photographed with a digital camera (Leica MC190 HD, Wetzlar, Germany). 

### 2.3. Molecular Characterization

The species-specific primers, SCAR markers, and the ITS region were selected for molecular characterization of *Meloidogyne hapla* isolates found in the survey. The total DNA was extracted from a single juvenile using the DNeasy Blood & Tissue Kit (Qiagen, Hilden, Germany) following the manufacturer’s instructions. The SCAR region was amplified in multiplex PCR using the primers JMV1 (5′-GGATGGCGTGCTTTCAAC-3′), JMV2 (5′-TTTCCCCTTATGATGTTTACCC-3′) and JMVhapla (5′AAAAATCCCCTCGAAAAATCCACC3′), and the ITS region, using the forward primer, TW81 (5′-GTTTCCGTAGGTGAACCTGC-3′) and the reverse AB28 (5′-ATATGCTTAAGTTCAGCGGGT-3′) [22,23]. PCR reactions were performed in a 50-μL final volume mixture containing 25 µL of Supreme NZYTaq II 2× Green Master Mix (NZYTech, Lisboa, Portugal), 1 µL of isolated DNA and 0.2 µM of each primer in a Biometra TGradient thermocycler (Biometra, Göttingen, Germany). Thermal cycling conditions were as described by [23,24]. PCR products were resolved by electrophoresis at 5 V.cm^−1^, in agarose gel (1.5%) containing 0.5 µg/mL of ethidium bromide and 0.5× of Tris-borate-EDTA (TBE) running buffer. Amplifications were visualized using the VersaDoc Imaging System (BioRad Laboratories, Hercules, CA, USA). PCR products were purified using the DNA Clean & Concentrator Kit (Zymo Research Corp, Irvine, CA, USA), according to the manufacturer’s instructions. Amplicons were sequenced at the INIAV (Oeiras, Portugal) on an ABI PRISM 3730xl (Applied Biosystems, Walthman, MA, USA) DNA analyzer. The newly obtained sequences were manually checked, edited and assembled. The sequences were compared to those of *M. hapla* and other relevant sequences of *Meloidogyne* spp. available in the GenBank database using the BLAST homology search. The multiple alignment of the retrieved sequences was performed using ClustalW multiple alignment in BioEdit.

Phylogenetic analyses were conducted using MEGA 11 [25], the maximum likelihood (ML) and the Kimura 2-parameter model. The robustness of the ML tree was inferred using 1000 bootstrap replicates.

## 3. Results

### 3.1. Distribution

**Figure 1:**
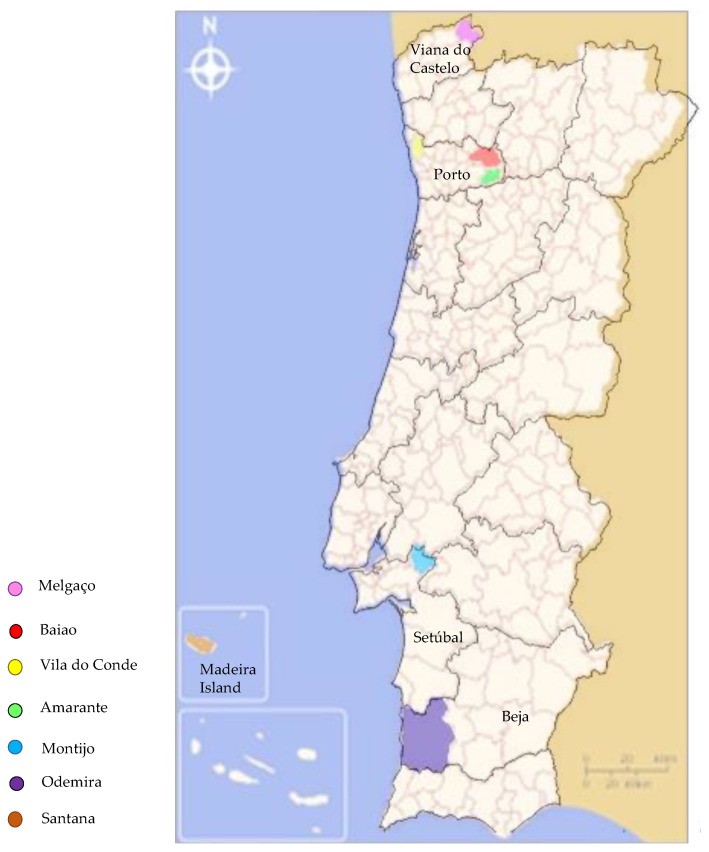
Counties and districts where *Meloidogyne hapla* was detected.

A total of 690 samples were collected from the south to the north of mainland Portugal and Madeira Island. From the total of samples collected, *Meloidogyne hapla* was detected in nine, corresponding to 1.5%. The detections were in four districts of the southern and northern regions and on Madeira Island. *Meloidogyne hapla* was not found in the central region. Three hosts were identified (potato, grapevine and eucalyptus) (Table 1) (Figure 1).

### 3.2. Symptoms

The field symptoms (above- and below-ground) observed and the species of nematodes found in the soil are described in Table 2.

**Table 2:** Symptoms observed in plants infected with *Meloidogyne hapla* and plant parasitic nematodes (PPNs) found in soil.

Crop	Above-Ground Symptoms (Figure 2)	Below-Ground Symptoms (Figure 3)	PPNs Present in Soil
Grapevine	Drying of the bunches Yellowing Poor growth	Presence of galls and egg masses	70 J2 of *Meloidogyne*/400 mL No other active PPNs were found in the soil extractions
Eucalyptus	Yellowing Stunting Dead plants	----	70 J2 of *Meloidogyne*/400 mL No other active PPNs were found in the soil extractions
Potato	Poor growth	Presence of galls and egg masses	80 J2 of *Meloidogyne*/400 mL No other active PPNs were found in the soil extractions

### 3.3. Morphological Characterization

**Figure 4:**
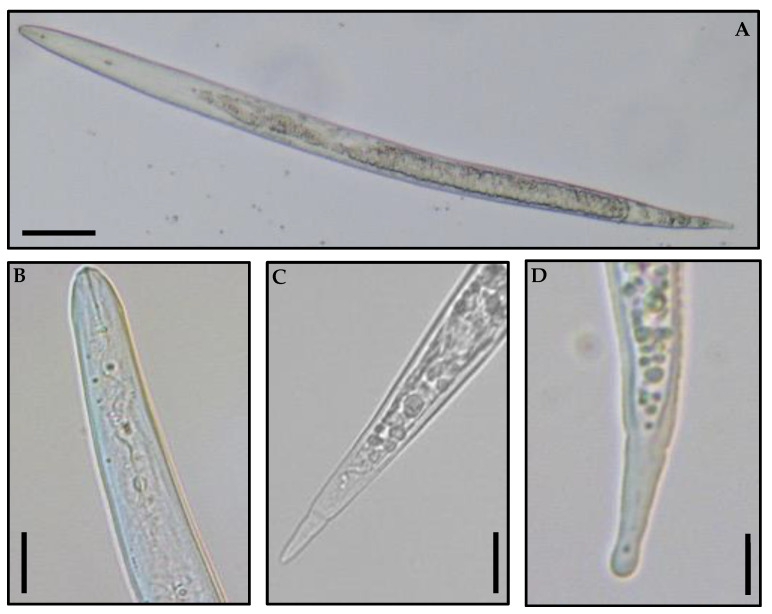
*Meloidogyne hapla* light microscope observations. Second-stage juvenile: (**A**) whole specimen; (**B**) anterior region; (**C**) tail region; (**D**) hyaline part (bar = 20 µm).

Morphological characterization from the recovered second-stage juveniles was performed and was in agreement with previous descriptions of the genus (Figure 4) [24,26,27]. The second-stage juveniles were vermiform, slender, and clearly annulated. The head region was slightly set off from body. The stylet was delicate and narrow, and the dorsal gland orifice (DGO) long, compared to the other three most common species. The knobs were rounded and appeared set off from the shaft. The excretory pore was distinct and hemizonid anterior or adjacent to excretory pore. The tail was long and slender with a narrow, tapering terminus that had several distinct annulations; the tail delimitation was not very clear in *M. hapla*. The hyaline tail terminus of *M. hapla* is highly variable and can go from deformed with an irregular shape to regular V shapes. 

### 3.4. Molecular Characterization

**Figure 5:**
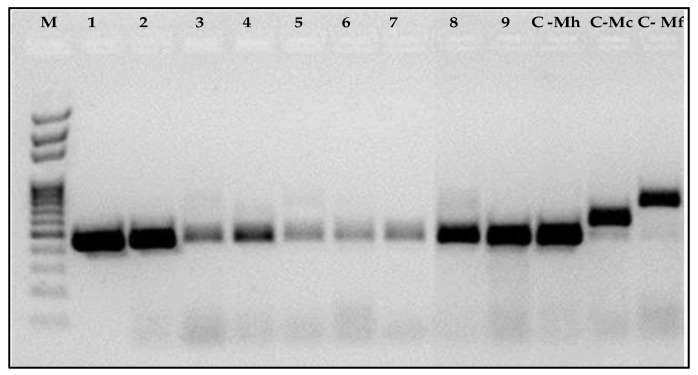
DNA amplification products obtained from isolates of *Meloidogyne hapla* using species-specific primers in multiplex (JMV1, JMV2 and JMVhapla). M: 100-bp DNA ladder (BIORON); Lanes 1–9: *M. hapla*. Positive controls: C-Mh: *M. hapla*; C-Mc: *M. chitwoodi*; C-Mf: *M. fallax*.

The PCR amplification of species-specific SCAR markers yielded a single fragment of 440 bp as expected (Figure 5). The amplification of ITS (TW81/AB28) yielded a single fragment of about 550 bp. The nucleotide sequences obtained in this study were deposited into the GenBank database (NCBI) under the accession numbers, OP364010, OP364011, OP364012, OP364013, OP364014, OP364015, OP364016, OP364017 and OP364018. A BLAST search of the nucleotide sequences showed a similarity ranging from 99.8% to 100% with the sequences of *M. hapla* available in the database (Table 3).

**Figure 6:**
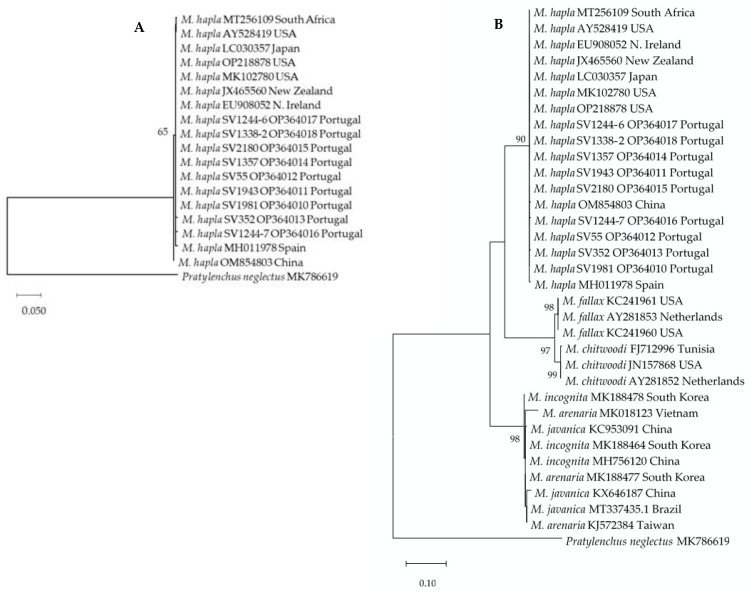
Phylogenetic relationships of *Meloidogyne hapla* isolates collected from Portugal, and *M. hapla* isolates from other geographical regions, including other species of the *Meloidogyne* group, based on the sequence alignment of the ITS region. The dendrogram was inferred by using the maximum likelihood method and the Kimura 2-parameter model. (**A**) This analysis involved 19 nucleotide sequences and there was a total of 261 positions in the final dataset. (**B**) This analysis involved 34 nucleotide sequences and there was a total of 261 positions in the final dataset. Evolutionary analyses were conducted in MEGA 11.

The molecular phylogenetic analysis is presented in Figure 6. The phylogram **A** revealed one clade, supported by a bootstrap value of 65%, that included only isolates of *M. hapla* from different hosts and geographical origins including Portugal. The phylogram **B** revealed three distinct clades: one represented by isolates of *M. hapla* from a different host and locations including Portugal, supported by a bootstrap value of 90%; a second one containing isolates of *M. chitwood* and *M. fallax* (bootstrap value of 97%); and a third clade with isolates of tropical species of *Meloidogyne* (bootstrap value of 98%).

## 4. Discussion

Nematodes are often overlooked because of the ambiguity of the symptoms they cause, and the difficulty in detecting and identifying them. Moreover, the impact the RKN has in agricultural areas reinforces the need for accurate diagnosis at the species level. Conventionally, the identification of *Meloidogyne* species has been based on morphological characters of second-stage juveniles, perineal patterns of adult females and isozyme phenotypes. Isozymes are highly reliable, but have its drawbacks, as it requires a specific developmental stage (adult females) as well as considerable skills. Many different DNA-based methods have been used to identify *M. hapla*, among which include species-specific SCAR primers and the ITS region; these methods allow to identify the species in a timely and efficient manner. Since the eradication of nematodes is a complex task, the identification of the *Meloidogyne* spp. in the area of interest is of primary importance to implement management strategies that minimize damage. This will allow for the adoption of preventive practices such as the appropriate deployment of cultivars. 

The presence of *M. hapla* has extended throughout the country, from detections in two districts in 2009 to five in the following four years (2019–2022). Such occurrences and increased detection draw attention to its potential to adversely affect economically important horticultural and ornamental crops. These detections were in northern and southern coastal counties—places where the climate is influenced by the Atlantic Ocean. Districts located up north are cooler and rainy, while the south becomes gradually warmer and sunnier; however, the Atlantic moderates the temperature, providing adequate conditions for *M. hapla* development and survival, as average temperatures throughout the year vary from 5 °C to 25 °C. 

To our knowledge, this is the first report of *Meloidogyne hapla* in the grapevine and eucalyptus in Portugal, adding valuable information to the current situation of this organism in the European Plant Protection Organization (EPPO) zone.

## 5. Conclusions

Root-knot nematodes are one of the most devastating plant parasitic nematodes, affecting the yield and quality of many crops. Their wide host range makes the management of RKNs extremely difficult; so, identifying the nematode species we are dealing with and understanding the spread, survival and damage potential, in this case of the *Meloidogyne hapla,* is the first step to managing it efficiently and effectively. 

This study confirmed the presence of *M. hapla* in the country and provided evidence of its dispersion and ability to survive in different crops, contributing important data that can be used in integrated management programs. 

## Figures and Tables

**Figure 2:**
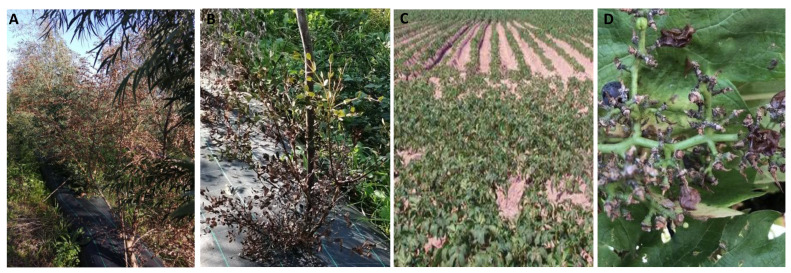
Above-ground symptoms of *Meloidogyne hapla* attack. (**A**,**B**): eucalyptus; (**C**): potato; (**D**): grapevine.

**Figure 3:**
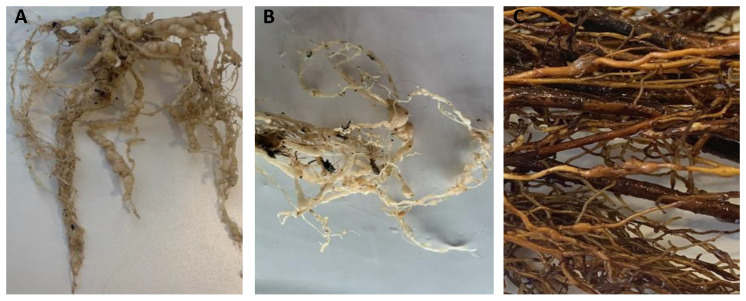
Below-ground symptoms of *Meloidogyne hapla* attack (galls). (**A**,**B**): potato; (**C**): grapevine.

**Table 1:** Locations, dates and crops where *M. hapla* was found.

Code	Year	District	County	Crop
SV1943	2019	Beja	Odemira	Grapevine
SV55	2020	Madeira Island	Santana	Grapevine
SV1981	2020	Beja	Odemira	Eucalyptus
SV1357	2021	Viana de Castelo	Melgaço	Potato
SV2180	2021	Porto	Vila do Conde	Potato
SV352	2022	Setúbal	Montijo	Grapevine
SV12446	2022	Porto	Amarante	Potato
SV12447	2022	Porto	Amarante	Potato
SV13382	2022	Porto	Baião	Potato

**Table 3:** Details of isolates obtained in this study, and isolates representing species of *Meloidogyne hapla* retrieved from GenBank and used in phylogenetic analyses.

Species	Isolate/Year	Country	Host	GenBank Accession
*M. hapla*	SV1943/2019	Portugal	Grapevine	OP364011
*M. hapla*	SV55/2020	Portugal	Grapevine	OP364012
*M. hapla*	SV1981/2020	Portugal	Eucalyptus	OP364010
*M. hapla*	SV1357/2021	Portugal	Potato	OP364014
*M. hapla*	SV2180/2021	Portugal	Potato	OP364015
*M. hapla*	SV352/2022	Portugal	Grapevine	OP364013
*M. hapla*	SV12446/2022	Portugal	Potato	OP364017
*M. hapla*	SV12447/2022	Portugal	Potato	OP364016
*M. hapla*	SV13382/2022	Portugal	Potato	OP364018
*M. hapla*	4179/2008	N. Ireland	Turf grasses	EU908052
*M. hapla*	n.a	Hawaii	Spanish needle	AY528419
*M. hapla*	Kiyokawa 1/2018	Japan	Tomato	LC030357
*M. hapla*	n.a	China	Carrot	OM854803
*M. hapla*	MH1/2021	India	Carrot	MZ964890
*M. hapla*	Limpopo/2020	South Africa	Kiwifruit	MT256109
*M. hapla*	F3-25/50/2022	USA	Strawberry	OP218878
*M. hapla*	RT83/2019	USA	Knockout Rose	MK102780
*M. hapla*	V70/2018	Spain	Wild olive	MH011978
*M. fallax*	824,Clone8/2014	USA	Turf grasses	KC241961
*M. fallax*	825,Clone7/2014	USA	Turf grasses	KC241960
*M. fallax*	n.a	Netherlands	Beetroot	AY281853
*M. chitwoodi*	728-1/2020	USA	Turf grasses	JN157868
*M. chitwoodi*	n.a	Netherlands	Wheat	AY281852
*M. chitwoodi*	PpKL5/2017	Tunisia	Fababean	FJ712996
*M. incognita*	SS62516/2019	South Korea	Pepper	MK188478
*M. incognita*	DW7336/2019	South Korea	Cucucmber	MK188464
*M. incognita*	GXM03/2018	China	Gynochtodes	MH756120
*M. arenaria*	GS0488/2019	South Korea	Melon	MK188477
*M. arenaria*	4564/2018	Vietnam	Black pepper	MK018123
*M. arenaria*	Tuku/2014	Taiwan	Peanut	KJ572384
*M. javanica*	XSBN-1/2016	China	Hemp	KX646187
*M. javanica*	XSYT01/2013	China	Hemp	KC953091
*M. javanica*	CN0027/2020	Brazil	Zinnia	MT337435

## Data Availability

Not applicable.
